# Reproductive strategy and gamete development of an invasive fanworm, *Sabella spallanzanii* (Polychaeta: Sabellidae), a field study in Gulf St Vincent, South Australia

**DOI:** 10.1371/journal.pone.0200027

**Published:** 2018-07-03

**Authors:** Aria L. Lee, Katherine A. Dafforn, Pat A. Hutchings, Emma L. Johnston

**Affiliations:** 1 School of Biological, Earth and Environmental Sciences, University of New South Wales, Sydney, NSW, Australia; 2 Department of Environmental Sciences, Macquarie University, North Ryde, NSW Australia; 3 Sydney Institute of Marine Sciences, Mosman, NSW, Australia; 4 Australian Museum Research Institute, Australian Museum, Sydney, Australia; Gettysburg College, UNITED STATES

## Abstract

Several reproductive strategies have been identified as key factors that contribute to the establishment and dispersal of invasive species in new environments. These strategies include early maturity, high reproductive capacity and flexibility in timing of reproduction. It is therefore critical to investigate the reproductive biology of target exotic species to understand their potential for population increase and invasive spread, and to inform management control strategies. The European fanworm, *Sabella spallanzanii* (Gmelin 1791), has established invasive populations along the southern coast of Australia. Gamete development and reproductive periodicity of this worm were investigated in two populations in Gulf St Vincent in South Australia over a 1 year period (July 2012 to June 2013). Samples of worms were collected monthly and dissected for histological analysis. Most individuals reached reproductive maturity at 70 mm body length (thorax and abdomen). Individuals from both populations contained mature and developing gametes year-round and a distinct spawning season was not observed. This may indicate sustained spawning by the population over the year, which provides a constant supply of new recruits to the area. Body length and egg size of worms from these populations were smaller than conspecifics in its native range and other invasive locations. Reproduction and development of *S*. *spallanzanii* differs not only between native and invasive locations, but also within invasive locations. This study has shown that *S*. *spallanzanii* exhibits a higher phenotypic plasticity and reproductive flexibility than previously known.

## Introduction

The reproductive biology of a species provides insight into population dynamics and ecological function [1–3]. As humans become more influential in driving ecosystem change, an understanding of the reproductive strategies of key species is crucial for the effective and sustainable management of natural resources [4]. One group that requires intense focus in this regard is invasive pests, organisms that are known to proliferate rapidly and impact upon ecosystems [5]. Poor understanding of life history characteristics of many marine pests hampers our capacity to prevent invasions and minimise spread, and there remain large gaps in our understanding of life-history characteristics of many marine pests.

Differing reproductive strategies have evolved to optimise the dispersal and survival of offspring [6–8]. The strategic variation in gamete quality, size and quantity affect offspring fitness including growth, reproduction and survival [9–11], particularly for species with non-feeding larvae [12–14]. Characteristics of rapid colonisers include increased growth rates, short generation times and mechanisms for wide dispersal [15,16]. Such mechanisms include broadcast spawning, which is a common strategy for reproduction and dispersal used by marine organisms, including pest species [17]. Synchronised broadcast spawning is commonly triggered by environmental conditions such as temperature, light intensity, or chemical cues [18,19]. The timing of gamete release for broadcast spawners is crucial to ensure maximum fertilisation of ova, and to aid in larval dispersion [20]. However, flexibility in the timing of these spawning events can allow species to overcome environmental or inter- and intra-specific challenges to achieve a greater reproductive gain.

One group of successful marine invaders are the sabellid polychaetes. Many of these tube-dwelling species have become widespread invasive pests, including *Euchone limnicola* in Australia [21]; *Terebrasabella heterouncinata* in abalone farms worldwide [22]; and several species of *Branchiomma* in the Mediterranean, east coast of South America and west coast USA [23,24]. The diversity of sabellids also extends to their methods of reproduction [25,26]. Sabellids may exhibit intratubular fertilisation and brood larvae within and outside their tubes, external fertilisation by broadcast spawning, as well as asexual reproduction by fission [27–30]. Such diversity in reproductive strategies allows different sabellid species to populate new environments and contribute to the invasive success of this family. This diversity also means that we must understand the unique reproductive traits of invasive sabellid species targeted for management.

There have been few studies detailing the reproductive periodicity of sabellid species. Smaller species have been found to be intratubular brooders that reproduce for extended periods or continuously [28,31,32]. Most larger species studied exhibited synchronous broadcast spawning that occurred yearly over a period of 2–3 months [33–35]. Of the invasive sabellids studied, only *Sabella spallanzanii* reproduction has been investigated in both its native and introduced locations.

The European fanworm, *S*. *spallanzanii* has a widespread native distribution in the Mediterranean Sea and the east Atlantic coast to the southern end of the English Channel [29]. It was first found in Australian waters in 1965 [36], and has established viable populations along the southern coast of Australia [37,38]. Phylogeographic analyses of Australian populations found low genetic diversity suggesting that the species had probably been translocated from one or two areas in the Mediterranean [39]. This species has also been introduced to New Zealand [40], imported from the Australian population [39]. Benthic populations of *S*. *spallanzanii* have been found to affect the oxygen, nitrogen and nutrients cyclings in the sediment [41]. It has been declared one of ten top priority marine pest in Australia [42] and is known to reduce the recruitment and community composition of a range of co-occurring invertebrates [43,44]. This species favours nutrient rich water at depths of 0.5–30 m, and can settle on hard substrate, or on rocks or shell in soft sediment. A gregarious settler, *S*. *spallanzanii* can grow in dense patches of up to 300 individuals per square metre [45], with their tubes providing substrate for epifaunal species, as well as refuge for macroinvertebrates [43,46].

Previous studies investigating *S*. *spallanzanii* reproduction have been done in its native Ionian Sea, Italy [47,48], and in Port Phillip Bay, Australia, where it is invasive [49]. These studies found that *S*. *spallanzanii* are broadcast spawners, exhibiting intratubular fertilisation. Sexes are separate, with a sex ration of 1:1. Gametes are formed from peritoneal cells in the coelomic cavity of abdominal sections, where they develop before release. Larvae are lecithotrophic and can survive for up to 21 days before settlement, the longest known for any sabellid [47]. In the Ionian Sea, it was found that spawning coincided with falling sea temperatures and concluded when the yearly minimum sea temperature was reached. Male and female spawning was largely synchronous. The Port Phillip Bay population varied slightly in the months of spawning over the austral autumn and winter months. Gametogenesis and development periods also differed, as did worm length at reproductive maturity. Such variability between studies suggests that the findings cannot reliably be applied to *S*. *spallanzanii* populations in other geographic areas.

Management of the distribution and spread of this species is dependent on reliable knowledge of its reproductive life history [40,50]. This study aimed to describe the reproductive biology of *S*. *spallanzanii* populations in Gulf St Vincent, South Australia. This species was first recorded in this area in the early 1990s [51], but there have been no previous attempts to record their reproductive periodicity. Gamete development and reproductive periodicity was assessed over the course of 1 year by histological analysis. Worm size at maturity, gamete sizes and worm morphology were examined. In particular, this study focused on describing changes in monthly gamete abundance to identify the time of year that these populations have the highest reproductive potential. Understanding the timing of reproduction can enable managers to identify a period within the life cycle of this species where management strategies will be most effective.

## Methods

### Field site and sample collections

**Fig 1:**
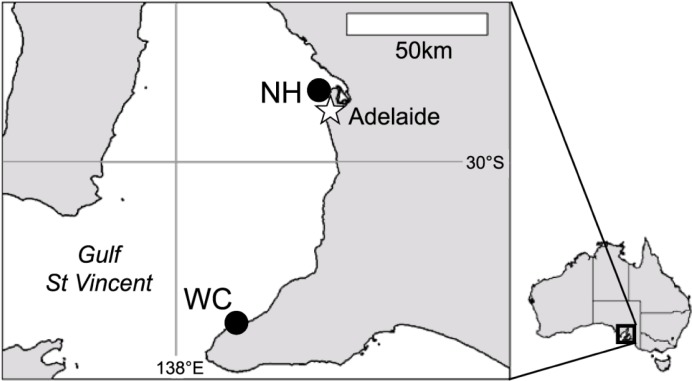
Study location. Field sites at North Haven (NH) and Wirrina Cove (WC) in Gulf St Vincent, South Australia.

To assess the reproductive biology of the invasive fanworm, *Sabella spallanzanii*, samples were collected from marinas at two sites, Wirrina Cove (35° 30’5”S, 138°14’38”E), hereafter WC, and North Haven harbour (34°47’9”S, 138°29’15”E), hereafter NH, on the south-eastern shore of Gulf St Vincent, South Australia (Fig 1). WC is located in a rural area of South Australia and houses a 270 berth marina for recreational vessels. NH lies within the boundaries of suburban Adelaide and contains several marinas and wharves to berth over 500 vessels for recreational and commercial use. Marinas at both sites are partially enclosed by artificial breakwaters. At each site, *S*. *spallanzanii* individuals were collected at the end of each month for a 1 year period from July 2012 to June 2013. Individuals were collected by hand by divers from the pilings and floating wharves of the marina at 0.5–1 m below MLWS. Specimen collection was authorised and conducted by Biosecurity SA, a department of the Government of South Australia. Individuals with tube length >50 mm were considered reproductively mature [49] and 20 worms in this size category were retained from each month of sampling for further processing. Crown, thorax and abdomen lengths were recorded for each individual before preservation in 10% formalin solution in seawater.

### Histological analysis

Histological analysis following Currie et al. (2000) was used to determine sex and to assess the reproductive potential of *S*. *spallanzanii* over time. Serial transverse sections of each individual were taken from the abdomen, beginning three chaetigers from the end of the thorax, ending 10 mm from the tail. Transverse sections were taken by cutting cross-section blocks (5 mm wide) at four equal intervals along the abdomen of each individual. Each block was then dehydrated in ethanol, mounted in paraffin wax and sectioned at 7μm. Four transverse sections (1 section per block) per individual were mounted on slides and stained with haematoxylin and eosin. The section with the highest density of gametes was taken for further analysis to represent the maximum reproductive potential of an individual. Sex was then determined by visual examination under a Leica DMLB compound light microscope (Germany) by identifying the presence of either egg or sperm in the coelomic cavity. For this study, individuals containing no gametes were classified Indeterminate Sex (IS) and excluded from gamete analysis.

### Female reproductive potential

Reproductive potential of female specimens was measured by counting gametes within three random sub-sections of the coelomic cavity of each transverse section, to calculate the mean number of eggs per mm^2^ of coelom. Three digital images (666 x 500 μm) were captured of each cross-section using a compound light microscope at 20x magnification, covering a total area of 0.999 mm^2^. Ova length was measured at its widest axis and assigned to one of three size classes (modified from Currie et al. (2000). Developing eggs (<50 μm) consisted of a basophilic nucleus, surrounded by a round, darkly stained eosiniophilic cytoplasm, where no distinct vitellin deposits could be differentiated from the cytoplasm. Early mature eggs (50–100 μm) exhibited a lightly stained yolk surrounded by a small amount of cytoplasm. Late mature eggs (>100 μm) consisted of larger amounts of cytoplasm when compared with yolk size.

### Male reproductive potential

Male reproductive potential was assessed by counting the number of sperm within three random sub-sections of the coelomic cavity of each transverse section, to find the estimated mean number of sperm per mm^2^ of coelom. Three digital images (120 x 90 μm) were captured using a compound light microscope at 100x magnification, covering a total area of 0.043 mm^2^. Sperm cells in each sub-section were counted using CellProfiler image software[52] and classified into two size classes (modified from Currie et al. [49]) according to widest axis length: 1.5–2.5 μm (mature), 2.5–10 μm (developing).

### Environmental data

Water temperature measurements were taken at both sites at the time of each monthly sample using a TPS sonde field logger (TPS Pty Ltd, Australia). At each site, three temperature recordings (at the water surface, 2 m depth and seafloor) were taken at the time of each monthly sampling. The mean of the temperature readings per month at each site was used as an environmental indicator of *S*. *spallanzanii* reproductive potential.

### Data analysis

Data analyses were conducted using R (version 3.4.1). Generalised Linear Models (GLMs) were used to estimate the effects of month on each egg and sperm size class and post-hoc Tukey pairwise tests were used to determine differences in gamete numbers between contiguous months. A gaussian distribution was used based upon examination of the residual vs fitted plots. Equivalence testing was used to test for differences in temporal gamete trends between males and females and assess reproductive synchronicity. GLMs using a negative binomial distribution were run using the *MASS* package [53] to test the effect of sex and month, and sex and month with an interaction with sex and month, on gamete abundance. A likelihood ratio test was then conducted between the models to determine the differences between gamete abundance over month when controlling for sex. Only data from the Wirrina Cove site was used for analyses and data from North Haven was excluded from these analyses due to missing data at three time points.

## Results

The histological staining of *S*. *spallanzanii* sections successfully highlighted reproductive features in 301 of the 480 dissected worms which comprised 174 females (116 WC; 58 NH) and 127 males (67 WC; 60 NH). Forty individuals (WC only) lacked gametes and were classified as Indeterminate Sex (IS). IS individuals were found throughout the year. Due to sampling constraints at NH, preservation of female worms was unsuccessful in August and September, and preservation of males worms was unsuccessful in November.

**Fig 2:**
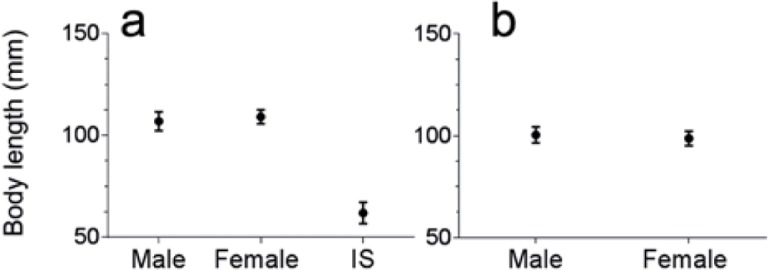
Body length of *Sabella spallanzanii*. Mean (±SE) worm body length (thorax and abdomen) from male, female and Indeterminate Sex (IS) specimens collected over 12 months at (a) Wirrina Cove and (b) North Haven.

At Wirrina Cove, female mean body length was 109 ± 3.4 (SE) mm; male mean body length was 107 ± 4.6 SE) mm; IS mean body length was 62 ± 5.3 (SE) mm (Fig 2A). At North Haven, female mean body length was 99 ± 3.6 (SE) mm; male mean body length was 99 ± 4.3 (SE) (Fig 2B). At 70 mm body length most individuals, male and female, exhibited gametes in their coelom (Fig 2).

## Gamete analysis

**Fig 3:**
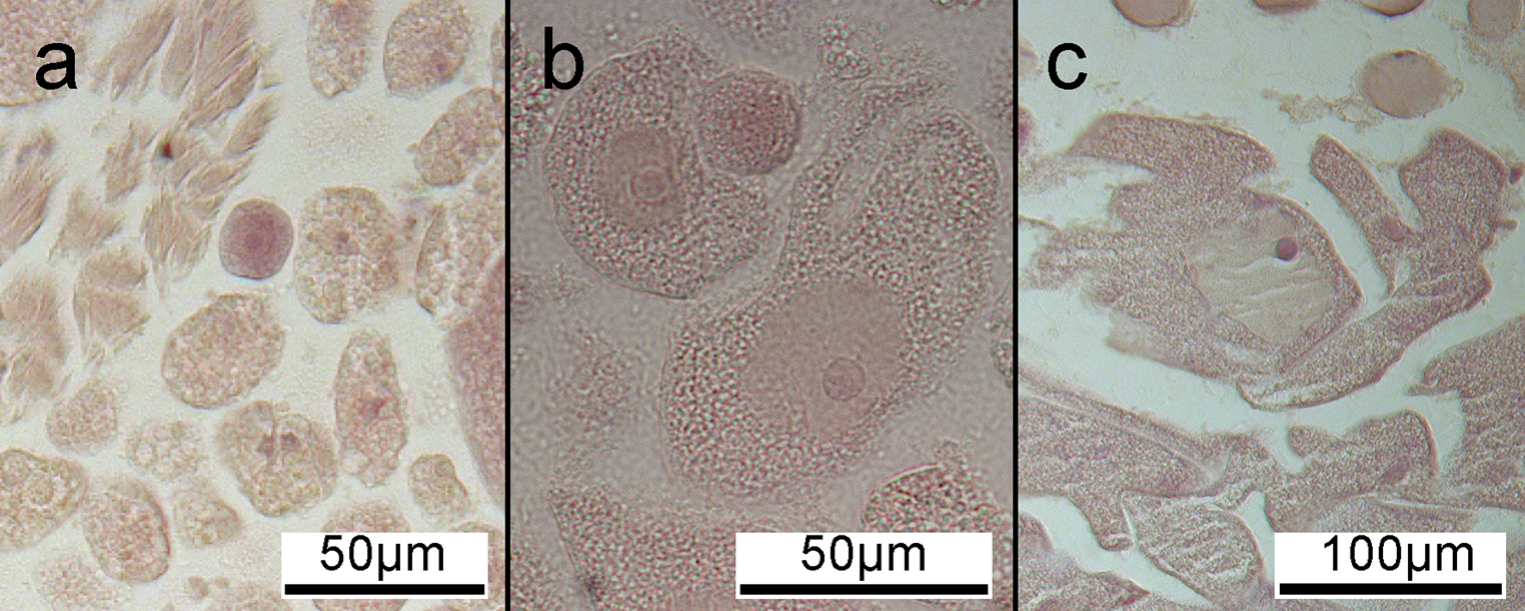
Female gametes. Transverse abdominal sections of female *Sabella spallanzanii* specimens showing eggs: (a) small developing oocyte (centre, pink); (b) early mature eggs displaying distinct yolk sac surrounded by cytoplasm; (c) late mature egg showing increased mass of yolk and cytoplasm.

Ova were dispersed throughout the coelomic cavity of female specimens. The smallest oocytes identified were 10 μm diameter and the largest observed egg measured 170 μm diameter (Fig 3).

**Fig 4:**
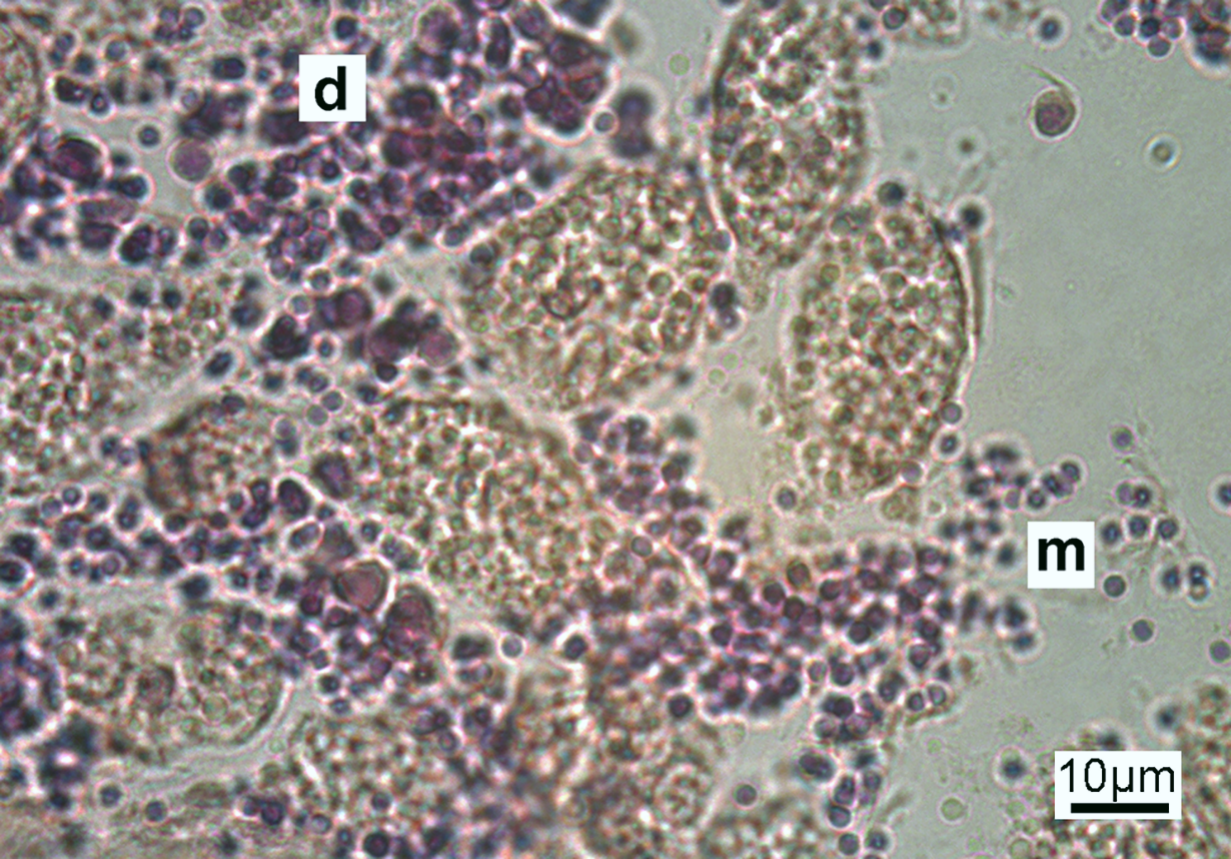
Male gametes. Transverse abdominal section of male *Sabella spallanzanii* specimen showing developing (d) and mature sperm (m).

Sperm cells were observed in dense patches within each transverse section of male specimens. Developing spermatids were oval shaped ranging between 2.5–10 μm diameter, distinct from mature sperm which were circular in shape, between 1.5–2.5 μm diameter (Fig 4).

### Reproductive potential

**Fig 5:**
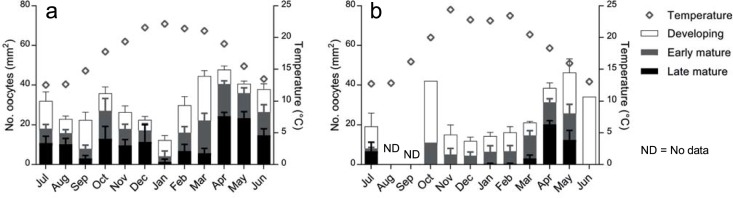
Female gamete abundance. Mean (±SE) oocyte numbers from female specimens from July 2012 to June 2013 from (a) Wirrina Cove and (b) North Haven, in three size classes: developing (<50 μm); early mature (50–100 μm); late mature (>100 μm). Values are stacked.

**Fig 6:**
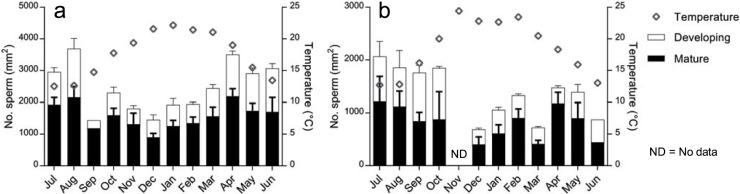
Male gamete abundance. Mean (±SE) sperm numbers from male specimens from July 2012 to June 2013 from (a) Wirrina Cove and (b) North Haven, in two size classes: developing (2.5–10 μm); mature (1.5–2.5 μm). Values are stacked.

Gamete abundance varied at all stages of development for males and females. The greatest density of oocytes occurred in April at WC (mean = 48 mm^-2^) and May at NH (mean = 38 mm^-2^) (Fig 5), while the greatest density of sperm occurred in August at WC (mean = 3688 mm^-2^) and July at North Haven (mean = 2065 mm^-2^) (Fig 6). Gamete abundances for both males and females were lower in the austral summer months. The peak in yearly temperature coincided with an increasing trend of gamete abundance in males and females at Wirrina Cove, while this pattern was not as consistent at North Haven. Though gamete abundance varied temporally (Table 1), there was no significant difference between adjacent months to indicate a spawning event (pairwise, p >0.05, S1 Table). The likelihood ratio from equivalence testing models showed no evidence of synchronicity of gamete abundance between males and females (χ^2^(11) = 10.25, p > 0.05) (S2 Table).

**Table 1:** Summary of GLMs of the effects of month on gamete abundance at Wirrina Cove. Significant p-values (p < 0.05) indicated in bold. Pairwise tests of adjacent months in S1 Table.

		Df	Deviance	P-value
Female	total	11	10974.22	**0.001**
	developing	11	2882.51	**0.003**
	early mature	11	2195.19	**0.006**
	late mature	11	5670.30	**< 0.001**
Male	total	11	95739365	0.090
	developing	11	56925177	0.248
	mature	11	13624317	**0.000**

## Discussion

The reproductive biology and life history stages of an invasive species is an important consideration for managers seeking to implement population controls or eradication [4,40,54]. We investigated size and gamete density of individuals of the invasive fanworm *Sabella spallanzanii* in Gulf St Vincent, South Australia, over a period of twelve months. We found that individuals from both sites exhibited a high degree of variation in the density of gametes over time, but importantly mature gametes were present continuously throughout the year in both sexes. Contrary to previous studies [45,49](Table 2), we suggest that spawning is not seasonally limited but can occur year-round with potential peaks as waters warm. This has implications for the timing of management efforts, such as culling or eradication attempts, and could inform maintenance practices in the marinas that harbour this invasive species.

**Table 2:** Comparison of *Sabella spallanzanii* in Gulf St Vincent to previous studies in the Ionian Sea, Italy [47], and Port Phillip Bay, Australia [49].

	Ionian Sea	Port Phillip Bay	Gulf St Vincent
Egg size at maturation (width)	250 μm	160 μm	50–170 μm
Sperm size at maturation (width)	2 μm	2 μm	2 μm
Spawning onset	late autumn/early winter	autumn/winter	early summer (potential peak spawning event)
Sea temperature at main spawning event	14 / 11°C	14 / 11°C	21 / 20°C
Worm body length at reproductive maturity	150 mm	50 mm	60–90 mm

Marine species that reproduce by broadcast spawning generally adopt temporally restricted or synchronous spawning to maximise fertilisation success [20]. However, both male and female individuals in this study carried mature gametes in every month of sampling, giving them the capacity to contribute to reproduction throughout the year. Previously, *S*. *spallanzanii* was found to spawn over a period of 2–3 months, with males and females spawning synchronously [45](Table 1). However, the opposite strategy may be advantageous for a population that is in a favourable area, such as an enclosed harbour. Marina breakwalls reduce flow rates and contains gametes, allowing additional opportunity for fertilisation and larval settlement [55].

Although marinas are hotspots of metal contamination [56] that may reduce polychaete reproductive success [57], there are also areas that concentrate nutrients and food for new recruits and are generally associated with higher rates of biofouling [58]. In such artificial environments the advantages of synchronous spawning could be nullified and a population that has a sustained larval supply throughout the year may be advantaged. Species like *S*. *spallanzanii* with lecithotrophic larvae that do not rely on planktonic food availability especially benefit from year-round spawning. Multiple small spawning events may increase the likelihood of settlement and colonisation of introduced species relative to less frequent large pulses as larvae can take advantage of heterogeneous availability and scare resources such as settlement space [59,60].

Though there is great variation within and between months, the abundance of mature gametes in both marinas decreased in December and January indicating a potential spawning peak immediately prior. The level of variability in gamete abundance that we observed is consistent with that recorded for the population of Port Phillip Bay. However the trend of decreased abundance in this bay occurred earlier, during September [49](Table 1 It is worth noting that the individuals studied from Port Phillip Bay were collected from locations near the mouth of the Bay where the environment is subjected to unrestricted water movement. However, the inner shores of the Bay (>25 km from mouth) exhibit a more stable nutrient enriched environment that may present suitable reproductive conditions year-round, and *S*. *spallanzanii* recruitment has been reported during the summer months in these locations [61]. The patterns of gamete abundance in these invasive locations are in stark contrast to the patterns observed for populations in their native locations in the Mediterranean. In their native range, worms were observed with no gametes within the coelom for 3 months followed by a 6 month period where gametes matured, and ended with a month of spawning where almost all gametes were released from the coelom [45]. The ability for *S*. *spallanzanii* to extend its spawning period in its non-native locations enables a longer supply of propagules for colonisation, increasing the potential for this species to increase in abundance.

Reproductive flexibility is found in many invasive species at the fringes of their distribution range. Flexibility allows for adaptation to local conditions that aid in range expansion [62]. Gametes that reach maturity more quickly enable rapid colonisation of a new area. This is particularly relevant for marine organisms that rely on broadcast spawning to reproduce. The largest observed ovum in Gulf St Vincent measured 170 μm diameter; however, it is unclear at what size ova in this population are fully mature. Ova sizes were considerably smaller when compared with the minimum egg size at maturation of 250 μm in the Ionian Sea, and 160 μm in Port Phillip Bay. This may be a result of the trade-off between quality and number of gametes to ensure maximum fitness [63]. Smaller eggs from lecithotrophic species have been shown to develop more quickly [64]. In a marina environment that is partially enclosed, the ability to quickly recruit and settle can rapidly build the population. Similarly, the ability to produce a large number of gametes can facilitate rapid population expansion.

Furthermore, *S*. *spallanzanii* in invasive populations have been found to be morphologically different than in native populations. This high level of phenotypic plasticity has been found among invasive species living in ideal conditions [65]. Additionally, *S*. *spallanzanii* shares traits common to successful invaders including smaller body size [66,67], and early sexual maturity [68]. In its native range, *S*. *spallanzanii* measures 150 mm body length at maturity [47]. In Gulf St Vincent, smaller individuals were found with mature gametes. At 70 mm body length many worms contained gametes, and few worms over 90 mm body length were observed without any gametes. This size range is consistent with the invasive population in Port Phillip Bay, where reproductive maturity was observed at 50 mm body length [49].

Morphological and reproductive differences such as these may in some instances be explained by cryptic speciation, which is common in sabellid species, however, we found no evidence that the population in Gulf St Vincent contains cryptic species complexes. With the largest body size of the sabellid polychaetes, *S*. *spallanzanii* has clear species markers in their crown and thoracic morphology. Additionally, genetic analyses by Ahyong et al. [39] of *S*. *spallanzanii* from Australia (including Gulf St Vincent), New Zealand and Europe showed very low genetic diversity between geographic regions. It is therefore very unlikely that the variation in our results is due to cryptic species complexes.

### Management implications

The role of marinas and harbours in facilitating the spread of non-indigenous species is well established [69–72]. Boat hull and pontoon cleaning regimes are unregulated, relying on guideline that users can opt to follow [73,74]. Cleaning regimes consist predominantly of manually removing fouling assemblages, and these disturbance events can trigger the release of gametes. It is essential to carefully consider of the time of year to implement these regimes so as not to facilitate spawning and increase the infestation [60]. In terms of lowest mature gamete abundance in Gulf St Vincent, the southern hemisphere summer would be ideal for manual removal. However, larval supply and settlement patterns of *S*. *spallanzanii* would also need to be considered. Control plans generally attempt to remove colonisers prior to spawning but must not trigger spawning due to disturbance [75]. Though chlorine solution biocides to eliminate adults have been trialled in New Zealand [76], its effect on spawning or larval survival was not examined.

The ability to adapt to local environmental conditions makes it difficult for managers to predict the severity and time frame for an incursion in a new location. Sabellid polychaetes exhibit a wide range of intraspecific plasticity in their morphology and reproductive ability [26,77]. *Sabella spallanzanii* exhibits many differences in reproductive periodicity and morphology that indicate that populations are adapted to local environmental conditions. The adaptability of this species and the potential for it to reproduce year-round highlights the need for bespoke research, management and monitoring programmes in harbours and coastal invasion hotspots.

## Supporting information

S1 Table Results of post-hoc Tukey’s pairwise tests between adjacent months from GLMs of the effects of month on gamete abundance at Wirrina Cove (Table 1). (XLSX)

S2 Table Summary of GLMs of the effects of Sex and Month on gamete abundance. (XLSX)

S1 Dataset *Sabella spallanzanii* in Gulf St Vincent. (CSV)
